# Diagnostic values of history taking, physical examination and KT-1000 arthrometer for suspect anterior cruciate ligament injuries in children and adolescents: a prospective diagnostic study

**DOI:** 10.1186/s12891-022-05659-1

**Published:** 2022-07-26

**Authors:** Martijn Dietvorst, M. C. Marieke van der Steen, Max Reijman, Rob P. A. Janssen

**Affiliations:** 1 Department of Orthopedic Surgery and Trauma, Máxima MC, Eindhoven, the Netherlands; 2 Department of Orthopedic Surgery and Trauma, Catharina Hospital Eindhoven, Eindhoven, the Netherlands; 3 Department of Orthopedic Surgery, Erasmus University MC, Rotterdam, the Netherlands; 4 Department of Biomedical Engineering, Orthopedic Biomechanics, Eindhoven University of Technology, Eindhoven, the Netherlands; 5 Department of Paramedical Sciences, Chair Value-Based Health Care, Fontys University of Applied Sciences, Eindhoven, the Netherlands

**Keywords:** Anterior cruciate ligament injuries, Paediatric, Adolescent, Diagnostics, History taking, Stability tests, Arthrometer

## Abstract

**Background:**

Diagnosing anterior cruciate ligament (ACL) injuries in children and adolescents are more challenging compared to adults. Delayed diagnosis may result in meniscal or chondral injuries. The aim of this study was to determine the diagnostic values of history taking, physical examination and KT-1000 arthrometer for suspect ACL injuries in children and adolescents.

**Methods:**

In this prospective diagnostic study, all children and adolescents (< 18 years) with post-traumatic knee complaints presenting at the out-patient department of the Máxima MC were eligible for inclusion. One experienced knee specialised orthopaedic surgeon was blinded and performed history taking, physical examination and KT-1000 arthrometer measurement. All patients had a magnetic resonance imaging (MRI) for the final diagnosis. Diagnostic values of interest were sensitivity, specificity, positive and negative predictive values (PPV and NPV). The outcomes of the KT-1000 arthrometer were drafted in a relative operating characteristics (ROC) curve to determine the optimal cut-off points.

**Results:**

Sixty-six patients were included, of which 50 had an ACL rupture and 16 had no ACL rupture on MRI. Report of a popping sensation during trauma had a specificity and PPV of 100% for diagnosing ACL injuries. The PPV and NPV of the Lachman test (in case of describing end-feel) were 95 and 82%, of the anterior drawer test 87 and 90% and of the pivot shift test 95 and 81% respectively. The optimal cut-off point of the KT-1000 arthrometer at 133 N force was an absolute translation of ≥7 mm with a PPV and NPV of 97 and 88% respectively.

**Conclusions:**

Report of a popping sensation during trauma has a specificity and PPV of 100% for diagnosing ACL injuries in children and adolescents. Although potentially difficult in children, the Lachman test, anterior drawer test and pivot shift test have a high PPV and NPV when performed by an experienced orthopaedic surgeon. An absolute anterior translation of ≥7 mm of the injured knee in the KT-1000 arthrometer at 133 N has the highest diagnostic values of all tests for diagnosing ACL injuries.

**Level of evidence:**

3

## Background

Anterior cruciate ligament (ACL) ruptures are a severe injury of the knee for children and adolescents [1]. Paediatric ACL tears are rare, accounting for less than 5% of all ACL injuries, and rarely occur under the age of 9 [1, 2]. Management of paediatric ACL injuries is challenging and a matter of debate due to limited scientific evidence [1, 3]. Diagnosing an ACL injury in children is also more challenging compared to adults [1]. This may be due to difficulty in obtaining an accurate history, greater physiological joint laxity and lack of cooperation during physical examination [1, 4, 5]. Besides, skeletally immature children may sustain different knee injuries than adults, such as an epiphysiolysis or sleeve fracture of the patella, and the frequency of specific injuries is different within adolescent age categories [1, 6]. A missed or delayed diagnosis and treatment of an ACL rupture in children and adolescents increase the risk of -irreparable- meniscal lesions or chondral lesions [7–11]. Besides, a false positive diagnosis might result in unnecessary referrals to orthopaedic surgeons and magnetic resonance imaging (MRI).

There is currently limited evidence on the diagnostic values of history taking and physical examination in children as there are no prospective, diagnostic studies on this topic. In their retrospective study, Kocher et al. [4] determined the diagnostic accuracy of the physical examination in children with intra-articular disorders of the knee necessitating an arthroscopic evaluation, including ACL, meniscal and chondral injuries [4]. The sensitivity and specificity of the physical examination for diagnosing an ACL injury were respectively 81.3 and 90.6% [4]. History taking and ACL injury specific tests, such as the Lachman test, anterior drawer test, pivot shift test or the KT-1000 arthrometer have not been evaluated for diagnosing ACL injuries in children and adolescents specifically [4, 12–16].

The aim of this study is to determine the diagnostic values of a standardized history taking, physical examination and KT-1000 arthrometer for suspect ACL injuries in children and adolescents. It is hypothesized that children and adolescents report similar anamnestic items as adults, but due to greater physiological laxity and paediatric specific injuries, the diagnostic values of physical examination are lower than those reported in literature for adults.

## Methods

### Study design

In this prospective diagnostic study, all children and adolescents (< 18 years) with post-traumatic knee complaints presenting consecutively between 2017 and 2021 at the out-patient clinic of an experienced knee specialized orthopaedic surgeon (RJ) were eligible for inclusion. There were no restrictions on time interval between trauma and consultation nor on referral. Patients who had undergone surgical treatment for the knee complaints or for whom the reason for referral was known were excluded. The orthopaedic surgeon was blinded for the reason of referral and for prior diagnostic outcomes including physical examinations, radiographs and MRI’s. A standardized history taking and physical examination including the KT-1000 arthrometer was performed by the orthopaedic surgeon. All patients had an MRI of the affected knee before or after the consultation.

### Tests

A complete history taking on the trauma and post-traumatic complaints was performed in all patients. As this study aims at diagnostic values of history taking for suspected ACL injuries, three common symptoms were registered [17, 18]:

- Q1: Did you experience a popping sensation in the knee during trauma?
- Q2: Was there acute post-traumatic effusion of the knee?
- Q3: Do you have complaints of instability (giving way) of the knee?

During history taking, patients were invited to explain all aspects of their complaints, symptoms and injury mechanism by open questions. Specific details such as a popping sensation, post-traumatic effusion and/or feeling of instability were additionally asked if not previously mentioned.

Physical examination was performed in a standard manner according to International Knee Documentation Committee (IKDC) 2000 knee ligament standard evaluation form [1, 19, 20]. Tests of interests were the Lachman test, anterior drawer test and pivot shift test [1, 19, 20]. An increased translation compared to the contralateral side was defined as a positive outcome. After the physical examination, the orthopaedic surgeon indicated for whether the patient was suspect for an ACL injury based on the outcomes of history taking and physical examination.

After the physical examination, the KT-1000 arthrometer was used to quantify the anterior-posterior laxity of both knees. The KT-1000 arthrometer is an objective instrument that measures anterior tibial translation relative to the femur and is often used as dichotomous outcome tool, but not as continuous outcome variables [21]. The KT-1000 arthrometer is strapped to the leg, the tibia is pulled anteriorly with 67 N, 89 N and 133 N and the amount of anterior translation (mm) is measured [21]. Anterior translation of the injured and non-injured knee was measured and compared. The anterior translation of the target knee, measured in mm at 133 N force, was the outcome of interest, as normative data of this force are published in children and adolescents [22]. Outcomes at 67 N and 87 N were also gathered in order to compare the diagnostic capacities of the three different forces. Unfortunately, the KT-1000 arthrometer was only available at one of the two out-patient departments in which the orthopaedic surgeon was consulted.

### MRI

The MRI was used as a reference test, as an MRI is less invasive than arthroscopy and the sensitivity and specificity for detecting ACL injuries are 95 and 88% in children and adolescents [23]. All patients had an MRI of the knee before or after the consultation. The outcomes of the MRI were analysed by a radiologist who was not aware of the study. ACL injuries defined as full and partial ACL ruptures on MRI were the primary outcome of interest, in addition other ligament, meniscal and chondral injuries or fractures were recorded. Outcomes of the MRI were compared to outcomes of surgery on ACL injuries and meniscal injuries, in case when the patient did not have another trauma in between the MRI and surgery.

## Statistical analyses

Baseline characteristics were calculated for the study population. Sensitivity, specificity, positive predictive values (PPV) and negative predictive values (NPV) including the 95%-confidence intervals (95%-CI) were calculated as diagnostic values for the questions during history taking and the laxity tests during physical examination. Moreover, the final judgement on suspicion of ACL injury after history taking and after physical examination was evaluated. Clinically relevant diagnostic pathways based on the PPV and NPV were illustrated. The diagnostic values were calculated for the KT-1000 arthrometer. The outcomes were drafted in a Receiver Operating Characteristic (ROC) curve and the Area Under the Curve (AUC) was calculated for the KT-1000 arthrometer at 67 N, 87 N and 133 N. The Youden index were calculated for absolute and relative (difference between injured and control leg) anterior translations. Differences in anterior tibial translation between patients legs were calculated with the Wilcoxon signed rank test and between ACL injured and non-ACL injured children with the Mann-Whitney U test. Differences between complete and partial ACL injured children were calculated for history taking and physical examination based on cross tabs and Chi square, Fisher’s exact test or linear-by-linear association.

Based on preliminary results of this study on the prevalence of ACL injuries, the requirement of minimal sample size was calculated based on Bujang et al. [24]. Based on the prevalence of 70–80% and prior published sensitivity and specificity of diagnostic tests [25], the minimum sample size was calculated to consist of 60 patients, of which 48 patients having an ACL injury [24]. Due to the population size and skewness of data, medians and interquartile ranges were used to describe the central tendency. Statistical analyses were performed by using SPSS (version 22.0.0, IBM, Chicago, IL, USA). Significance was set at *p* = 0.05.

## Ethical approval

The study is approved by the local ethical committee of Máxima Medical Centre [N.17.020]. All included participants and their parents or legal guardians (if necessary) gave informed consent.

## Results

Sixty-seven patients were eligible for inclusion. One patient gave no consent for participation and was therefore excluded. The baseline characteristics of the 66 included patients are shown in Table 1. Age ranged from 7 to 17 years. All patients had post-traumatic knee complaints after a sports injury. 76% of the patients had an ACL injury. Of the 66 patients, 26 patients had a MRI before consultation, for which the orthopaedic surgeon was blinded. All other patients had a MRI after the consultation. All ACL injuries on MRI were confirmed during arthroscopy and all patients who underwent surgery for other indications had an intact ACL. There were 14 meniscal injuries diagnosed during ACL reconstruction, of which 8 meniscal injuries were prior diagnosed on MRI. Mean interval between MRI and ACL reconstruction for patients with meniscal injury was 186 days.

**Table 1:** Baseline characteristics of the study population

	Patients (*n* = 66)	
Age, y medians [IQR]	14	[12.5–15]
Gender, n female (%)	34	(52)
BMI, kg/m^2^ medians [IQR]	19.7	[17.7–22.3]
Open physes, n (%)	41	(62)
Previous knee complaints, n yes (%)	7	(11)
Time since trauma, weeks median [IQR]	22	[8–55]
Type of trauma, n (%)		
Non-contact	48	(73)
Contact	16	(24)
Missing	2	(3)
Injuries, n (%)^a^		
***ACL injuries***	***50***	***(76)***
Complete ACL rupture	45	(68)
Partial ACL rupture	5	(8)
***Other injuries***		
MCL injuries	1	(2)
LCL injuries	1	(2)
PCL injuries	1	(2)
Patellar dislocations	2	(3)
Meniscal injuries	12	(18)
Cartilage injuries	4	(6)
Osgood Schlatter	1	(2)
No injuries	9	(14)
Referral, n (%)		
General physician	24	(36)
Emergency department	8	(12)
Second opinion^b^	34	(52)

*ACL *Anterior Cruciate Ligament, *BMI *Body Mass Index, *IQR *Interquartile Range, *LCL *Lateral Collateral Ligament, *MCL *Medial Collateral Ligament, *MRI *Magnetic Resonance Imaging, *PCL *Posterior Cruciate Ligament. ^a^injuries diagnosed with history taking, physical examination or imaging. ^b^from orthopaedic surgeons or sports medicine doctors

### History taking and physical examination

**Fig. 1:**
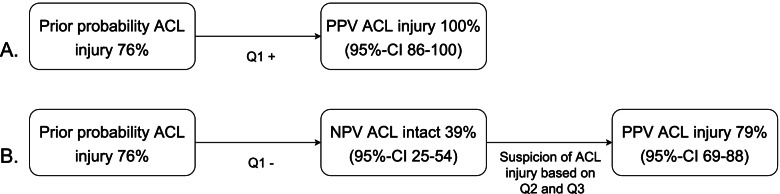
Different diagnostic pathways based on a popping sensation. **A** PPV of a positive popping sensation during history taking. **B** PPV of suspicion of ACL injury after history taking despite no popping sensation during trauma. “ACL = Anterior Cruciate Ligament”; “CI = Confidence Interval”; “NPV = Negative Predictive Value”; “PPV = Positive Predictive Value”

The diagnostic values of the questions (Q) during history taking are shown in Table 2. With the information gathered during history taking the orthopaedic surgeon misclassified 8 of 66 patients, resulting in a PPV of 89% and NPV of 83%. A popping sensation during trauma has a PPV of 100% and therefore diagnosis of an ACL injury is certain in case of a popping sensation. The NPV is 39% however, no popping sensation does therefore not rule out an ACL injury. Different diagnostic pathways are shown in Fig. 1 based on having experienced a popping sensation during trauma.

**Table 2:** Diagnostic values of the questions during history taking

	TP	FP	TN	FN	Sensitivity % (95%-CI)	Specificity % (95%-CI)	PPV % (95%-CI)	NPV % (95%-CI)
**Q1: Popping sensation**^**a**^	24	0	16	25	49 (35–63)	100 (81–100)	100 (86–100)	39 (25–54)
**Q2: Acute effusion**	49	12	4	1	98 (92–100)	25 (9–49)	80 (69–89)	80 (37–99)
**Q3: Instability (giving way)**	46	5	11	4	92 (82–97)	69 (45–88)	90 (80–96)	73 (49–91)
**Suspected for ACL injury**^**b**^	48	6	10	2	96 (88–99)	63 (38–83)	89 (79–95)	83 (57–97)

*ACL *Anterior Cruciate Ligament, *CI *Confidence Interval, *FN *False Negative, *FP *False Positive, *NPV *Negative Predictive Value, *PPV *Positive Predictive Value, *Q* Question, *TN* True Negative, TP True Positive

^a^in one case the popping sensation was not described; ^b^suspicion of ACL injury after history taking

Outcomes during history taking were similar between complete and partial injured children. Complaints of instability were however significantly different, as 96% of the children with a complete ACL injury had complaints of instability compared to 50% of the children with a partial ACL injury (*p* = 0.045).

**Fig. 2:**
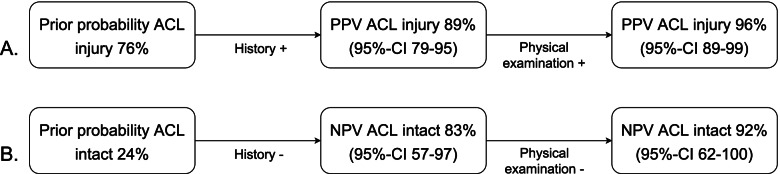
Different diagnostic pathways based on the suspicion after history taking and physical examination on ACL injuries. **A** PPV based on the suspicion of ACL injuries after history taking and physical examination. **B** NPV based on no suspicion of ACL injuries after history taking and physical examination. “ACL = Anterior Cruciate Ligament”; “CI = Confidence Interval”; “NPV = Negative Predictive Value”; “PPV = Positive Predictive Value”

Diagnostic values of the physical examination tests are shown in Table 3. In 6 patients the end-feel during Lachman test was not described and in one patient the results of the anterior drawer test were not described. The pivot shift could not be performed in 9 patients due to pain or problems in relaxation. After gathering information from both history taking and physical examination the orthopaedic surgeon correctly classified 63 of the 66 patients, as is shown in Fig. 2.

**Table 3:** Diagnostic values of laxity tests

	TP	FP	TN	FN	Sensitivity % (95%-CI)	Specificity % (95%-CI)	PPV % (95%-CI)	NPV % (95%-CI)
**Lachman test**	49	10	6	1	98 (92–100)	38 (17–62)	83 (72–91)	86 (51–99)
**Soft end-feel during Lachman test**	41	2	14	3	93 (83–98)	88 (66–98)	95 (86–99)	82 (60–95)
**Anterior drawer test**	48	7	9	1	98 (91–100)	56 (32–78)	87 (76–94)	90 (63–99)
**Pivot shift test**	39	2	13	3	93 (83–98)	87 (62–96)	95 (86–99)	81 (58–95)
**Suspected for ACL injury**^**a**^	49	2	14	1	98 (90–100)	88 (64–98)	96 (87–99)	93 (70–99)

*ACL *Anterior Cruciate Ligament, *CI *Confidence Interval, *FN *False Negative, *FP *False Positive, *NPV *Negative Predictive Value, *PPV *Positive Predictive Value, TN True Negative, *TP* True Positive

^a^suspicion of ACL injury after history taking and physical examination

Outcomes of Lachman test, end-feel, anterior drawer test and pivot shift test were significantly different in complete ACL injured versus partial ACL injured children. Lachman and anterior drawer were 6-10 mm or > 10 mm in 98 and 82% of the complete injured children compared to 25 and 25% of the partially ACL injured children (*p* = .001 and *p* = .039). All partially injured patients had a hard endpoint during Lachman test, compared to 2% of the complete ACL injured children (*p* = .000). None of the partially ACL injured children had a pivot shift of more than glide (1+). 78% of the completely ACL injured children had a pivot shift of clunk (2+) or more (*p* = .004).”

### KT-1000 arthrometer

A total of 40 patients were examined with a KT-1000 arthrometer at 133 N, of which 21 patients were also tested at 67 N and 87 N force. Of the 26 patients who were not examined with the KT-1000 arthrometer, 7 patients were not able to tolerate the KT-1000 arthrometer due to pain (5 patient with ACL injury and 2 patient without ACL injury). The legs of 3 patients were too small to fit in the KT-1000 arthrometer (2 patients with ACL injury and 1 without ACL injury) and 16 patient could not be examined with the KT-1000 arthrometer due to absence of the KT-1000 arthrometer at the location of the out-patient department visit (11 patients ACL injury and 5 patients without ACL injury). Group of partial ACL injuries was too small for subsequent analysis. Median anterior translations in the KT-1000 arthrometer are shown in Table 4.

**Table 4:** Median anterior translations of the tibia in the KT-1000 arthrometer at 133 N for the injured and control leg for patients with and without ACL injuries

	Median anterior translation of the tibia in mm [IQR]		***P***-value
	ACL injuries (***n*** = 32)	No ACL ruptures (***n*** = 8)	
**Injured leg**	10 [8.3–12.0]	5.0 [2.0–5.8]	>.001
**Control leg**	5.0 [3.0–7.0]	5.0 [2.0–5.0]	0.361
***P-*value**	>.001	1.000	

*ACL* Anterior Cruciate Ligament, *IQR *Interquartile Range, *mm *millimetres, *N *Newton

**Fig. 3:**
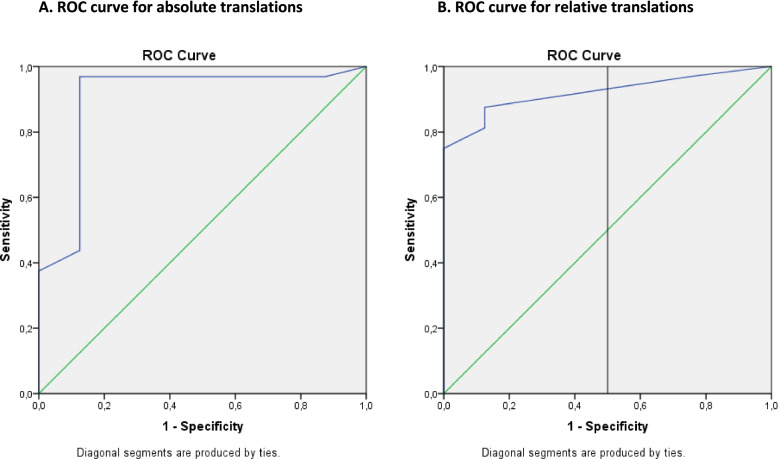
Receiver Operating Characteristics (ROC) curve for outcomes the KT-1000 arthrometer at 133 N for absolute anterior translations of the injured leg (**A**) and relative translations (**B**)

The diagnostic performance of the KT-1000 arthrometer at 133 N is shown in Fig. 3 for the absolute translation for the injured leg (3.1) and relative translation (difference between legs) (3.2). The AUC of the KT-1000 arthrometer at 133 N for relative translations was higher (0.973) compared to measurements at 67 N (0.953) and 87 N (0.947). The AUC for absolute translations were for 67 N, 87 N and 133 N respectively 0.920, 0,947 and 0,920. The Youden indexes at 133 N were higher than at 67 N and 87 N. Based on the Youden index, there is one optimal cut-off point for the absolute translation of the injured leg and two cut-off points for the relative translation, as is shown in Table 5. A relative translation of ≥1 mm resulted in a slightly lower PPV, but higher NPV compared to the cut-off of ≥4 mm.

**Table 5:** Optimal cut-off points of absolute and relative anterior tibial translation in the KT-1000 arthrometer at 133 N

Anterior tibial translation in millimetres (mm)		Sensitivity% (95%-CI)	Specificity% (95%-CI)	PPV % (95%-CI)	NPV % (95%-CI)	Youden Index
**Absolute (injured leg)**	7	97 (84–99)	88 (53–98)	97 (84–99)	88 (53–98)	0.84
**Relative (injured - control leg)**	1	88 (72–95)	88 (53–98)	97 (83–99)	64 (35–84)	0.75
	4	75 (58–87)	100 (68–100)	100 (86–100)	50 (28–72)	0.75

*CI* Confidence Interval, *mm * millimetres, *NPV *Negative Predictive Value, *PPV *Positive Predictive Value

## Discussion

The most important finding is that report of a popping sensation during trauma has a specificity and PPV of 100% for diagnosing ACL injuries in children and adolescents. The Lachman test, anterior drawer test and pivot shift test have a high PPV and NPV when performed by an experienced orthopaedic surgeon. An absolute anterior translation of ≥7 mm of the injured knee in the KT-1000 arthrometer at 133 N force is the most accurate test with a PPV of 97% and NPV of 88%. The KT-1000 arthrometer is therefore the most accurate clinical test to diagnose an ACL injury.

A missed or delayed diagnosis and treatment of an ACL rupture in children and adolescents increase the risk of an (irrepairable) meniscal lesion or chondral lesions [7–11]. Diagnosing an ACL injury in children however, can be more challenging compared to adults [1]. It is therefore important, especially in (primary) health care settings with a low prevalence of paediatric ACL injuries and professionals with limited experience in performing ACL stability tests, to screen for potential ACL injuries and to refer early to a specialized orthopaedic surgeon [14]. In adults, Geraets et al. [26] showed that for primary health care professionals only history taking is valuable when diagnosing ACL ruptures, while for orthopaedic surgeons diagnosis became more accurate when adding physical examination to medical history taking [26]. In the current study, report of a popping sensation resulted in a specificity and PPV of 100% with the 95%-CI of the specificity ranging from 81 to 100%. A report of a popping sensation did not necessitate additional examination to diagnose an ACL injury. A popping sensation is therefore a valuable diagnostic outcome for referral to an orthopaedic surgeon. However, careful physical examination is still essential as concomitant injuries might be present necessitating early treatment, such as a medial collateral ligament injury. Besides, the actual PPV in primary health care settings and general orthopaedic clinics might be lower as the prevalence of ACL injuries is lower [17].

In contrast to children and adolescents, the Lachman test, anterior drawer test and pivot shift test have been evaluated in an adult population before [25, 27]. In the most recent meta-analysis the Lachman and pivot shift tests showed a pooled sensitivity of respectively 89% (95%-CI 67–98%) and 79% (95%-CI 63–91%) in adults [25]. Compared to these diagnostic values in adults, the sensitivity of the Lachman test and pivot shift are higher in the current study. One would expect that the diagnostic values of tests would not be as high as in adults, due to specific issues in a paediatric and adolescent population, such as lack of patient cooperation and relaxation during examination, increased physiological laxity and the more varied differential diagnoses [1, 4, 5]. In the current study however, all tests were performed by a highly specialized orthopaedic surgeon in a setting with a high prevalence of ACL injuries in children. Experience level is a factor that might affect the reproducibility of the physical examination tests [25, 26]. This experience factor, combined with other factors, such as the size of the examiner hands, chronicity of the lesion and associated injuries, may also contribute to the variability of diagnostic values in literature [25, 26].

In order to objectify the anterior translation of the tibia, instrumented tests, such as the KT-1000 arthrometer, have been proposed to diagnose ACL injuries and to assess stability after ACL reconstruction [28]. In their systematic review and meta-analysis on the diagnostic values of arthrometers for diagnosing ACL injuries, van Eck et al. [28] found that the KT-1000 arthrometer at maximal manual force had the highest diagnostic values with a sensitivity of 93% and a specificity of 93%, although no cut-off value is presented [28]. The KT-1000 arthrometer is often used as dichotomous outcome diagnostic tool, measuring a difference of 2-3 mm between the legs (relative translation) [21]. The current study found that a relative translation at 133 N force had a greater AUC compared to 67 N and 87 N, although the AUC at 87 N was higher than at 133 N for absolute translations. The Youden indexes were highest for 133 N of force, these cut-offs were therefore used for analyses on diagnostic values. An absolute translation of ≥7 mm of the injured leg had a sensitivity of 97% (95-CI 84–100%) and a specificity of 88% (95%-CI 47–100%) and showed higher diagnostic values compared to cut-off values of relative translation (difference between injured and control leg). Interestingly, the often used cut-off values of a relative translation of 2 or 3 mm were not an optimal cut-off point in the current study, as both 1 mm and 4 mm relative translations had higher diagnostic values [21]. The absolute translation as a cut-off seemed to be most useful for diagnosing ACL injuries. However, absolute anterior translation should always be interpreted with caution as gender, pubertal growth phases and greater physiological joint hypermobility have influences on laxity and the contralateral leg should therefore always be evaluated [22, 29]. A limitation of KT-1000 arthrometer was that some children were too small to fit in the arthrometer or were unable to undergo the test due to fear or pain.

This study had certain limitations. The first limitation is that the MRI is used as reference test. Arthroscopy is regarded as the gold standard, but rarely used as diagnostic tool [30]. Diagnosis of ACL injuries on MRI is however highly accurate in children and teenagers and is less invasive, as was confirmed in the current study [23]. The MRI was therefore chosen as the reference test in the current study. Interestingly, half of the meniscal injuries were missed on MRI and found during ACL reconstruction in this study. The second limitation is that the Máxima Medical Centre is a tertiary referral centre for paediatric ACL injuries and the prevalence of ACL injuries was therefore high. This also resulted in a low prevalence of non-ACL injured patients, resulting in a relatively wide 95%-CI for specificity and NPV of some tests. Although the orthopaedic surgeon was blinded for the referral and MRI outcomes, one might expect that the high prevalence of ACL injuries potentially affected blinding as there was already a high suspicion of ACL injuries, especially when patients were referred from other hospitals. The third limitation was the population size of 66 children, which is smaller than some of the previous studies [4, 16]. However, the current study included a relatively large amount of children with ACL injuries and is the first study that evaluated history taking, ACL stability tests and KT-1000 m in this population. The population consisted of a few partially ACL injured children, which was a limitation in analysing differences between complete and partial ACL injuries. Final limitation was that joint hyperflexibility (for example Beighton scale) was not measured during physical examination. Previous study by Falciglia showed that KT-1000 laxity measurements were greater in adolescents with signs of physiological joint hyperflexibility [22].

## Conclusions

Report of a popping sensation during trauma has a specificity and PPV of 100% for diagnosing ACL injuries in children and adolescents. Although potentially difficult in children, the Lachman test, anterior drawer test and pivot shift test have a high PPV and NPV when performed by an experienced orthopaedic surgeon. An absolute anterior translation of ≥7 mm of the injured knee in the KT-1000 arthrometer at 133 N has the highest diagnostic values of all clinical tests for diagnosing ACL injuries.

## Data Availability

The datasets used and/or analysed during the current study available from the corresponding author on reasonable request.

## Abbreviations

ACL: Anterior Cruciate Ligament
AUC: Area Under the Curve
BMI: Body Mass Index
CI: Confidence Interval
FN: False Negatives
FP: False Positives
IKDC: International Knee Documentation Committee
IQR: InterQuartile Range
LCL: Lateral Collateral Ligament
MCL: Medial Collateral Ligament
mm: millimetres
MRI: Magnetic Resonance Imaging
N: Newton
NPV: Negative Predictive Value
PCL: Posterior Cruciate Ligament
PPV: Positive Predictive Value
Q: Question
ROC curve: Receiver Operating Characteristics curve
TN: True Negatives
TP: True Positives
